# Implementing integrative nursing for oncology inpatients: a retrospective analysis of project-related routine data from 2021 to 2023

**DOI:** 10.1007/s00520-026-10666-2

**Published:** 2026-04-17

**Authors:** Lea Raiber, Beate Stock-Schröer, Klaus Kramer

**Affiliations:** 1 Section Integrative Medicine, Department of General and Visceral Surgery, Ulm University Medical Center, Ulm, Germany; 2 Interprofessional Graduate School Integrative Medicine and Health, Health Department, Witten/Herdecke University, Witten, Germany

**Keywords:** Integrative oncology, Symptom burden, Supportive care, Symptom management, Nursing counseling, Non-pharmacological Interventions

## Abstract

**Purpose:**

The study explored the implementation of integrative nursing (IN) interventions in oncology inpatient care within a dedicated project. As part of an IN consultation service, trained integrative nurses delivered external naturopathic, non-pharmacological interventions. The aim of this study was to characterize patients receiving IN interventions and to describe how these interventions are implemented and applied in oncological inpatient care.

**Methods:**

This retrospective study analyzed routine project-related data collected at Ulm University Hospital between 2021 and 2023. Recorded variables included patient demographics, clinical characteristics, type and frequency of IN interventions, and immediate patient reaction. Quantitative data were analyzed descriptively, and qualitative data were examined using content analysis.

**Results:**

Healthcare professionals requested an IN consultation for 381 patients, of whom 361 (94.8%) agreed to participate. The majority were female (62.3%; *n* = 225) and between 60 and 69 years of age (33.5%; *n* = 121). In total, 1910 IN interventions were carried out, with a median of four IN interventions per patient (*M* = 5.3 ± 4.6; *r* = 1–30). Most IN interventions targeted the lower limbs (38.7%; *n* = 740), most frequently using rhythmic embrocation (70.9%; *n* = 1355) and solum oil (39.6%; *n* = 757). Immediately after the IN intervention, the most commonly observed patient reactions were relaxation (67.5%; *n* = 726) and deeper breathing (37.5%; *n* = 403).

**Conclusion:**

The high level of acceptance and the continuous increase in utilization suggest that IN was well implemented in clinical practice during the project, with positive short-term reactions from patients. Further intervention studies are needed to provide robust evidence of its efficacy and to support its long-term integration into routine hospital care.

## Introduction

Oncology patients experience a significant physical and mental burden: Study findings indicate that while the severity of symptoms was generally low at cancer diagnosis, the prevalence of symptoms such as fatigue, poor well-being, pain, and the need for supportive care was quite high [1]. More than one in two cancer patients experienced fatigue, low well-being, drowsiness, pain, and lack of appetite during an unplanned hospitalization, regardless of the type of cancer. Almost one in five patients exhibited relevant clinical symptoms of anxiety and depression [2]. Patients with advanced cancer have a high symptom burden during hospitalization, with more than one in two reporting moderate to severe symptoms (e.g., fatigue, pain, and drowsiness) [3]. Patients and healthcare professionals have identified the following symptoms as priorities for further research: fatigue, constipation, and diarrhea [4]. A high symptom burden in patients is associated on the one hand with a longer length of stay in hospital and on the other hand with a higher risk of unplanned hospital readmission [3]. The symptoms described have a negative impact on the quality of life of cancer patients [5, 6]. Newcomb et al. (2020) emphasize that supportive care interventions should aim to improve the patients’ symptoms [2]. Supportive care is defined as interventions aimed at preventing and managing the physical and psychological symptoms associated with cancer and its treatment, as well as supporting patients’ quality of life throughout the disease trajectory [7].

Integrative nursing (IN) describes a care approach that integrates conventional nursing with evidence-informed complementary interventions, aiming to address patients’ physical, emotional, social, and spiritual needs in a holistic manner [8–10]. IN is guided by six core principles, including whole-person care, the therapeutic nurse–patient relationship, the support of the body’s innate healing capacity, the healing potential of nature, the use of evidence-informed integrative therapies, and attention to the well-being of both patients and nurses [9, 11]. As an approach to supportive care, IN has the potential to benefit patients across various care settings by addressing symptom burden and promoting patient well-being [12–15].

The present study focuses primarily on the integrative symptom management, including a broad spectrum of external, naturopathic, non-pharmacological treatments such as compresses, rhythmic embrocations, and therapeutic washes and baths with essential oils. In recent years, an increasing number of evidence syntheses [16–18] and systematic reviews [12, 19–21] have been published in the field of complementary nursing therapies. The review by Mühlenpfordt et al. (2022) highlights the diverse potential of external nursing interventions in addressing a wide range of clinical indications [21].

Nevertheless, as shown in the systematic reviews, the overall study landscape remains heterogeneous, both in terms of reported outcomes and methodological quality. Furthermore, evidence on the implementation of IN in inpatient settings is scarce. To date, most research within the oncology context has primarily focused on pediatric patients [22–24] or on counseling in outpatient care [25–28]. Since 2021, IN has been gradually implemented across several wards at Ulm University Hospital within the project IMPLEMENT-UKU (“Implementation of IN at the Ulm University Hospital”). In this context, IN is provided as a consultation service for supportive care, with a particular focus on oncology inpatients.

Although IN is gaining increasing recognition, evidence on its feasibility and implementation as supportive care for oncology inpatients remains limited. Therefore, the primary aims of this study were to characterize the patients receiving IN and to describe the implementation and application of IN interventions in oncological inpatient care.

## Methods

### Study design and setting

A monocentric, retrospective cohort study was conducted among patients who participated in the IMPLEMENT-UKU project at Ulm University Hospital between 2021 and 2023. During this period, the project was implemented on up to four hospital wards. Data used in the present study were derived from project-related routine documentation and were collected through a retrospective chart review.

### Study population

The study sample comprised all patients who participated in the IMPLEMENT-UKU project between 2021 and 2023. The project included adult patients (≥ 18 years) for whom an IN consultation was requested by medical or nursing staff.

### IN in the project “IMPLEMENT-UKU”

As part of the IMPLEMENT-UKU project, oncology patients in participating wards received IN interventions as supportive care during their hospital stay through a consultation service. Patients were referred to the IN consultation service by nursing and medical staff when pertinent symptoms such as restlessness, pain, or cancer-related side effects required supportive care. Referred patients received an IN consultation followed by subsequent IN visits. Each IN visit consisted of three steps: first, an initial nurse-patient conversation guided by the 12 Activities of Daily Living according to Liliane Juchli’s nursing model and a person-centered conversation technique; second, the application of one or more symptom-oriented IN interventions; and third, a post-intervention rest period of up to 30 min. When necessary, patients were supported throughout their entire hospital stay until the time of discharge; preferably by a single integrative nurse, in order to provide a relationship-based care.

The IN interventions were delivered by integrative nurses who had completed 3 years of vocational training and possessed extensive professional experience. In addition, the nurses were specifically trained and held an additional qualification in external naturopathic interventions.

A comprehensive catalog of integrative therapies was developed to guide the IN interventions in clinical practice. The catalog included external naturopathic, non-pharmacological nursing treatments such as embrocations, compresses, wraps, and therapeutic washes and baths. Based on this catalog (Table 1), IN interventions were provided in addition to routine care, taking into account patients’ symptom burden and individual preferences. The catalog focused on cancer-related symptoms and served as a structured decision-support tool for integrative nurses when selecting symptom-oriented IN interventions. It was developed through an expert consensus process, drawing on textbooks and academic literature on external naturopathic interventions [17, 18, 29–33], as well as recommendations from existing projects and guidelines in the field of complementary nursing therapies [25, 34–39], considered within the context of IN [40–42]. To ensure intervention fidelity, standard operation procedures were established for all IN interventions.

**Table 1:** Catalog of IN interventions used in the IMPLEMENT-UKU project (as of August 2023)

Symptoms	IN interventions^1^
Appetite loss	• Liver compress with yarrow tea or oil^2^
Respiratory insufficiency, respiratory infections	• Sternum-compress with thyme oil^2^, plantago bronchial balm^3^ • Embrocation with thyme oil^2^, solum oil^3^, plantago bronchial balm^3^ • Diaphragm-compress with copper ointment^3^, thyme oil^2^, solum oil^3^
Sleep disorders	• Heart-compress with aurum-lavandula ointment^3^ • Hand-/foot-bath with lavender bath milk^3^ • Sternum-compress with lavender oil^3^ • Embrocation with lavender oil^2^, solum oil^3^, mallow oil^3^ • Liver compress with yarrow tea or oil^2^
Exhaustion, weakness	• Hand-/foot-bath with citrus bath milk^3^, rosemary bath milk^3^ • Liver compress with yarrow tea oil^2^ • Therapeutic wash with citrus bath milk^3^, rosemary bath milk^3^, solum oil^3^ • Embrocation with mallow oil^3^, solum oil^3^, rose oil^2^
Depressed mood	• Embrocation with mallow oil^3^, rose oil^2^, citrus oil^2^ • Therapeutic wash with citrus bath milk^3^ • Liver compress with yarrow tea or oil^2^
Crisis situation	• Hand-/foot-bath with lavender bath milk^3^ • Embrocation with rose oil^2^, solum oil^3^, lavender oil^2^ • Therapeutic wash with rosemary bath milk^3^
Constipation, meteorism, irritable bowel syndrome	• Abdomen-embrocation with fennel-caraway oil^2^, chamomile oil^2^, melissa oil^3^, oxalis ointment^3^ • Abdomen compress with chamomile oil^2^
Edema, congestion	• Compress with borago essence^3^, curd, arnica essence^3^ • Embrocation with rosemary oil^2^, solum oil^3^ • Therapeutic wash with citrus bath milk^3^
Polyneuropathy	• Embrocation with aconite oil^3^, solum oil^3^
Pain	• Embrocation with aconite oil^3^, solum oil^3^, arnica essence^3^ • Warm/cool compress
Nausea, vomiting	• Abdomen-compress with fennel-caraway oil^2^, chamomile oil^2^, melissa oil^3^ • Embrocation with fennel-caraway oil^2^, chamomile oil^2^, melissa oil^3^
Restlessness, anxiety, tension	• Heart-compress with aurum-lavandula ointment^3^ • Liver-compress with yarrow tea or oil^1^ • Wrist-compress with citrus oil^2^ • Embrocation with solum oil^3^, lavender oil^2^, rose oil^2^, mallow oil^3^ • Sternum-compress with lavender oil^2^, arnica essence^3^ • Hand-/foot-bath with lavender bath milk^3^

^1^Selected according to the patient´s symptom burden and preferences

^2^Essential oil, for embrocations with a high-quality oil as a carrier

^3^Ready-to-use product

Embrocation = rhythmic embrocation according to Wegman/Hauschka

### Data collection

Data were extracted from the routine project-related documentation of all requested IN consultations and visits conducted by integrative nurses, which were systematically recorded. Only previously documented information was used. Information bias was reduced by relying on these standardized records. All data were anonymized and managed in Excel by trained study staff. Variables collected for the retrospective analysis included sociodemographic variables (age, sex, and oncological diagnosis), details of IN consultation service (hospital department, year), IN visit (duration of the preliminary conversation, documented symptoms and complaints), IN interventions (body region, type, used substance, and duration), and reactions following the IN intervention (perception directly after the intervention, further perceived reactions during follow-up). During the study period, the project-related documentation was adapted and further developed, since some variables were added at later stages. The sample size varied depending on the variable considered.

### Statistical analysis

Descriptive statistics were used to characterize the quantitative data, including measures of central tendency and dispersion as well as absolute and relative frequencies. To maximize the use of available information, cases with missing values were not fully excluded from the analysis. Therefore, the sample size varied across specific analyses. The data analysis was performed using IBM SPSS Statistics version 29.0 (IBM Corp., Armonk, NY, USA). To analyze the qualitative data from the free-text notes, a structured qualitative content analysis was conducted using MAXQDA (VERBI Software, Berlin, Germany) [43].

### Ethical consideration

The present study was approved by the Ethics Committee of Ulm University, Germany (No. 256/24) and was conducted in accordance with the Declaration of Helsinki. All data were anonymized, stored securely, and password protected. The study was registered at the German Clinical Trials Register (DRKS00034989). The reporting of this study adhered to the Strengthening the Reporting of Observational Studies in Epidemiology (STROBE) guidelines [44].

## Results

During the study period, nursing or medical staff requested an IN consultation for 381 patients. Of these, 20 patients refused the offer of IN, resulting in an acceptance rate of 94.8%. The reasons for refusal fell into eight main categories (*n* = 25; multiple responses possible): (1) no need from the patient’s point of view (*n* = 5), (2) state of exhaustion or overload (*n* = 3), (3) organizational reasons (*n* = 3), (4) no reasons documented (*n* = 3), (5) language or communication barriers (*n* = 2), (6) refusal of physical contact (*n* = 2), (7) incapacity to give consent (*n* = 2), and (8) other reasons (*n* = 5).

### Patient characteristics

A total of 361 patients received IN interventions during the study period, and their data were included in the retrospective study analysis (Table 2). One third (33.5%; *n* = 121) of the patients were aged between 60 and 69 years, and 62.3% (*n* = 225) were female. Almost all patients (95.0%; *n* = 343) had a primary oncological diagnosis. The most prevalent cancers were hematological malignancies (49.6%; *n* = 179) and solid tumors (41.8%; *n* = 151).

**Table 2:** Patient characteristics and project realization (*n* = 361)

	Characteristics	*n*	%
Age group, years	18–29	17	4.7
	30–39	18	5.0
	40–49	44	12.2
	50–59	68	18.8
	60–69	121	33.5
	70–79	68	18.8
	80 and older	24	6.6
	No data	1	0.3
Sex	Female	225	62.3
	Male	136	37.7
Oncological diagnosis	Yes	343	95.0
	No	13	3.6
	No data	5	1.4
Type of cancer	Hematological malignancies	179	49.6
	Solid tumor	151	41.8
	Other	26	7.2
	No data	5	1.4
Hospital department	Dept. for Internal Medicine	309	85.6
	Dept. for Gynaecology	52	14.4
Year	2021	27	7.5
	2022	83	23.0
	2023	251	69.5

### IN consultation service

A total of 1343 IN visits were performed, with an average of 3.7 ± 3.1 per patient (Mdn = 3.0; range = 1–22). The mean duration of the preliminary conversation between the nurse and the patient during IN visits was 10.8 ± 2.8 min (Mdn = 10.0; range = 5–30). In addition, a total of 1,910 IN interventions were performed during these IN visits, with a mean number of 5.3 ± 4.6 (Mdn = 4.0; range = 1–30) per patient during the inpatient stay.

### Symptom burden

A variety of symptoms were reported, perceived, and noted by the nurse during the IN visits (Table 3). The most prevalent complaints (multiple responses possible) included depressed mood (56.5%; *n* = 719), psychological crisis situations (55.7%; *n* = 709), and reduced mobility (43.9%; *n* = 559).

**Table 3:** Symptom burden of patients at the IN visits (*n* = 1273; multiple answer possible)

Present symptomatology	*n*	%
Depressed mood	719	56.5
Crisis situations, psychological	709	55.7
Other	659	51.8
Mobility restriction	559	43.9
Restlessness, tension, strain	537	42.2
Respiratory insufficiency or infection, dyspnoea	503	39.5
Oedema, congestion	468	36.8
Rigidity, stiffness	442	34.7
Anxiety	422	33.2
Pain	380	29.9
Exhaustion, tiredness	239	18.8
Insomnia, sleep disorders, disturbed sleep	166	13.0
Polyneuropathy	129	10.1
Skin changes	131	10.3
Intestinal complaints such as diarrhea or constipation	127	10.0
Nausea, vomiting	105	8.3
Insufficient temperature sensation	50	3.9

A median of 5.0 symptoms per patient and IN visit was documented (*M* = 4.9 ± 2.4; range = 0–12). In 83.8% (*n* = 1099) of IN visits, patients exhibited three or more symptoms. The symptom burden remained consistently high, ranging from a median of 4.5 (*M* = 4.6 ± 2.8; range = 1–10) to 6.0 (*M* = 5.5 ± 2.5; range = 0–10) symptoms across IN visits with more than ten cases.

### IN interventions

Details of the IN interventions are presented in Table 4. IN interventions were predominantly applied to the lower limbs (38.7%; *n* = 740) and the back (26.0%; *n* = 497). The most prevalent types of IN interventions were rhythmic embrocation (80.0%; *n* = 1528) and compresses or wraps (14.4%; *n* = 275). With regard to the substances used, solum oil (39.6%; *n* = 757), aconite oil (11.1%; *n* = 212), and lavender oil or bath milk (10.5%; *n* = 200) were the most frequently employed. The duration of IN interventions ranged from 5 to 60 min, with a median of 15 min (M = 17.3 ± 8.1; *n* = 1782). Overall, the cumulative time spent providing IN interventions per patient amounted to a median of 90 min.

**Table 4:** Details of the IN interventions (*n* = 1910) carried out

		*n*	%
Body region	Feet/lower legs	740	38.7
	Back	497	26.0
	Arms/hands	168	8.8
	Legs	162	8.5
	Abdomen (belly, liver)	153	8.0
	Chest	136	7.1
	Other	54	2.8
Type of IN intervention	Rhythmic embrocation	1528	80.0
	Compress or wrap	275	14.4
	Therapeutic bath	60	3.1
	Therapeutic wash	34	1.8
	Other	13	0.7
Used substance	Solum oil	757	39.6
	Aconite oil	212	11.1
	Lavender oil/bath milk	200	10.5
	Citrus oil/bath milk	149	7.8
	Rose oil	97	5.1
	Aurum-lavandula ointment	92	4.8
	Yarrow tea infusion/oil	78	4.1
	Rosemary oil/bath milk	74	3.9
	Mallow oil	64	3.4
	Plantago bronchial balm	42	2.2
	Borage essence	33	1.7
	Chamomile oil	19	1.0
	Oxalis ointment	17	0.9
	Arnica essence	16	0.8
	Thyme oil	14	0.7
	No substance	14	0.7
	Melissa oil	12	0.6
	Other	20	1.1

### Reactions after the IN interventions

**Fig. 1:**
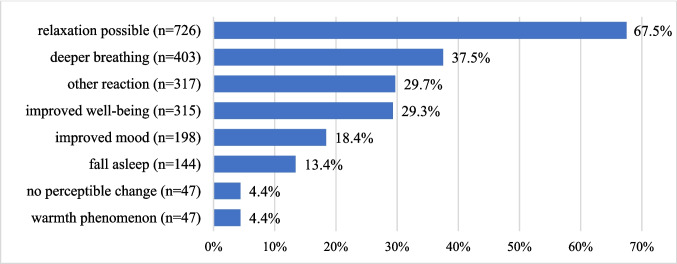
Perception of the nurse shortly after the IN intervention (*n* = 1076; multiple responses possible)

Immediately after the IN intervention, the most frequently observed patient reactions documented by nurses were possible relaxation (67.5%; *n* = 726), deeper breathing (37.5%; *n* = 403), and improved well-being (29.3%; *n* = 315). Multiple responses were possible. The findings are illustrated in Fig. 1.

After the initial observation, other observed reactions were documented by the integrative nurses. The free-text notes were assigned 384 codes across five overarching categories: (1) positive reactions (*n* = 299), (2) no or little perceptible reaction or persistent symptoms (*n* = 38), (3) reactions that could not be assigned to a specific category (*n* = 28), (4) other notes unrelated to the reaction (*n* = 11), and (5) negative reactions (*n* = 8). Positive reactions were associated with existing symptoms (*n* = 84), perceived emotional excitement (*n* = 94), and other positive effects (*n* = 121), including relief and sense of calm.

## Discussion

The present retrospective study analyzed the implementation of IN and the corresponding IN interventions within the IMPLEMENT-UKU project, conducted between 2021 and 2023 at a university hospital in Germany. The high acceptance rate of 94.8% observed during the initial three-year implementation phase suggests substantial patient interest in IN, and it underscores both its feasibility and its potential value as supportive care in routine clinical practice.

The study sample suggests a high demand for supportive care among oncology inpatients. Depressed mood and crisis situations, alongside other symptoms, were among the most frequently documented concerns, affecting nearly half of the patients. These findings align with prior research demonstrating that oncology inpatients experience a substantial physical and psychological symptom burden [2, 45, 46]. For instance, a German study revealed that almost every second hospitalized cancer patient reported elevated psychosocial distress [47]. A large-scale analysis of cancer patients revealed that fatigue, weakness, pain, and sleep problems play an important role alongside fear of progression [48]. While the referral process in the present study suggests a selection of patients with supportive care needs, the persistently high symptom burden emphasizes the importance of supportive care during hospitalization.

A total of 361 patients received 1910 IN interventions during the three-year implementation phase, with the number of interventions increasing steadily over time. A distinctive component of IN consultation services was the preliminary nurse-patient conversation, which was systematically integrated into each IN visit. Its duration varied according to individual needs, reflecting the heterogeneity of patients’ communicative and supportive requirements. This underscores the importance of relational and dialogical aspects as fundamental components of IN in patient-centered care [9, 11]. Consequently, symptom-oriented IN interventions could be determined on this basis.

Excluding preparation and documentation time, providing IN during hospitalization required a median of 90 min per patient. The effort per patient, however, varied considerably, largely depending on the length of hospital stay and individual needs. Building on this, the implementation of an IN consultation service requires substantial time resources, which must be carefully considered when planning and scaling such a service. In addition, the successful implementation of IN interventions requires the specialized training for integrative nurses, as this ensures their safe and effective application.

This retrospective analysis could not evaluate long-term effects or patient-reported outcomes; only nurses’ perceptions after the IN intervention were available based on routine data. The most frequently reported short-term reactions were relaxation and deeper breathing. Such immediate effects may be clinically relevant, as they can support symptom relief, stress reduction, and coping during hospitalization. Despite the limitation to nurse-reported outcomes, these findings are consistent with theoretical and empirical frameworks suggesting that tactile and mindfulness-based interventions may activate relaxation pathways, enhance parasympathetic regulation, and promote both emotional comfort and perceived well-being in patients [49–51].

This study provides initial insights into the successful implementation of IN in a university hospital setting using routine data. To support the sustainable integration of IN into routine inpatient care, future research should be guided by the principles of implementation science, addressing structural, organizational, and contextual factors. In addition, rigorously designed intervention studies are required to establish robust external evidence regarding the efficacy of IN interventions and to consolidate their role within evidence-based supportive cancer care.

### Limitations

The present study has several limitations. The study was conducted at a single center, and the absence of a control group limits the generalizability and consequently the external validity of the findings. As project-related routine data were documented by nursing staff, self-reported patient outcomes and validated patient-reported measures were not available. It is imperative that future studies are conducted to ascertain the efficacy of IN interventions in influencing clinical and patient-reported outcomes within the domain of oncology care. Due to its retrospective design, the analysis is subject to an inherent risk of bias. Although data were systematically collected within the project framework, information bias cannot be fully excluded, and unmeasured confounding could not be addressed. Selection bias was minimized, as all patients documented within the project during the study period were included without additional sampling. However, given that this was a consultation service, patients had already been selected when the request was made by staff on the ward. Finally, data quality was partially constrained by the presence of missing or incomplete variables in the project-related documentation, which may have introduced distortions.

## Conclusion

The present study provides insights into the implementation and application of IN interventions as supportive care in oncological inpatient care. The findings demonstrate the feasibility of integrating these interventions into routine clinical practice and their acceptance among patients in the hospital setting. Future prospective studies are needed to evaluate the impact of IN interventions on patient outcomes, including symptom burden, well-being, and quality of life, and to generate robust evidence supporting their sustained integration into routine hospital care.

## Data Availability

The data used and analyzed in this study are available upon reasonable request from the corresponding author.

## Abbreviations

*IN*: Integrative nursing
*IMPLEMENT-UKU*: Implementation of Integrative Nursing at the Ulm University Hospital
